# Beyond Reperfusion: Acute Ventricular Unloading and Cardioprotection During Myocardial Infarction

**DOI:** 10.1007/s12265-019-9863-z

**Published:** 2019-01-22

**Authors:** Jerry Curran, Daniel Burkhoff, Robert A. Kloner

**Affiliations:** 1 Abiomed, Inc, Danvers, MA USA; 2 Columbia University, New York, NY USA; 3 Huntington Medical Research Institutes, Pasadena, CA USA; 4 University of Southern California, Los Angeles, CA USA

**Keywords:** Mechanical Circulatory Support, Myocardial Infarction, Reperfusion Injury, Impella, Ischemia-reperfusion Injury, Hemodynamics

## Abstract

Heart failure is a major cause of morbidity and mortality around the world, and myocardial infarction is its leading cause. Myocardial infarction destroys viable myocardium, and this dead tissue is replaced by a non-contractile scar that results in impaired cardiac function and a significantly increased likelihood of the patient developing heart failure. Limiting infarct scar size has been the target of pre-clinical and clinical investigations for decades. However, beyond reperfusion, few therapies have translated into the clinic that limit its formation. New approaches are needed. This review will focus on new clinical and pre-clinical data demonstrating that acute ventricular unloading prior to reperfusion by means of percutaneous left ventricular support devices reduces ischemia-reperfusion injury and limits infarct scar size. Emphasis will be given to summarizing our current mechanistic understanding of this new therapeutic approach to treating myocardial infarction.

## Introduction

Despite recent advances in medical and device-based therapies, heart failure (HF) is a major cause of morbidity and mortality and is a significant socioeconomic burden [1]. It affects nearly 6 million people in the USA with approximately 550,000 new cases diagnosed each year. These numbers are projected to increase 46% by 2030, exacerbating the already epidemic scale of the disease [1]. Coronary artery disease and acute myocardial infarction (AMI) are the largest contributors to HF, accounting for over 65% of all cases [1, 2]. Each year, approximately 560,000 new cases of AMI are reported [3]. Recent data indicate that 25% of patients will develop HF within 1 year of their first AMI with 75% developing HF within 5 years [4, 5]. Prevention of AMI-dependent HF represents a significant opportunity to curb the HF epidemic.

During AMI, coronary blood flow through one or more arteries becomes severely limited or altogether stopped. Tissue downstream of the occlusion is deprived of oxygen and nutrients. If perfusion to this region is not rapidly restored, then myocardial cell death will follow. This dead muscle is slowly replaced by a non-contractile fibrotic scar which reduces ventricular contractile state. Indeed, scar size is proportional to post-AMI mortality [6–8]. Reduced heart function results in decreased blood pressure and cardiac output (CO) which initiates a cascade of neurohormonal activation, vasoconstriction, and salt and water retention aimed at maintaining CO and end-organ perfusion. However, these compensatory mechanisms also increase ventricular volume, filling pressures, wall stress, and myocardial oxygen demand. As a result, the mechanical and metabolic load on the remaining myocardium is increased. This persistent exposure to stress leads to chamber dilation, myocardial hypertrophy, cardiac fibrosis, apoptosis, attrition of myocardial capillary density, and a host of molecular changes, collectively referred to as ventricular remodeling. While initially compensatory, ventricular remodeling is a maladaptive process and is fundamental to the pathogenesis of heart failure. Halting or slowing ventricular remodeling is the therapeutic target in the management of HF patients [9]. Highlighting the importance of limiting the initial ischemic damage, a recent study of > 2600 patients treated with primary reperfusion demonstrated that for every 5% increase in infarct scar size, the 1-year all-cause mortality increases by 19%, and 1-year HF hospitalization increases by 20% [10].

It follows then that the most effective approach to abate the development of HF post-AMI is to develop treatments that minimize MI scar formation and prevent ventricular remodeling. To date, timely reperfusion is the only intervention clinically demonstrated to limit infarct scar formation. The maxim that “time is muscle” has led to near universal adoption of a door-to-balloon (DTB) time (defined as the time from first electrocardiogram confirming AMI to mechanical reperfusion) of 90 min as a metric of successful healthcare delivery, and achieving this metric has proven effective in promoting good patient outcomes [11]. Timely reperfusion has driven the 30-day mortality rate for AMI down from nearly 30 to just under 5% [12, 13]. However, current data indicate further decreasing DTB time will not likely yield additional benefits. Menees et al. [13] demonstrated that over the last decade even though national DTB times continuously and significantly fell, the survival rate over the same period remained the same. Paradoxically, as more and more patients are surviving the index AMI, there is a greater incidence of post-infarct left ventricular (LV) dysfunction and HF [5]. This review will discuss how acute ventricular unloading during AMI and prior to reperfusion may provide a new therapeutic approach to limiting infarct scar size.

## Limits of Reperfusion Therapy

As a therapy, reperfusion is, to a certain extent, self-limited because it independently can cause injury that is thought to include myocardial death. This detriment of reperfusion, termed ischemia-reperfusion (I-R) injury, is well-documented. Some investigators believe that up to 40–50% of final infarct scar size is due to damage upon reperfusion [14–18]. Yet, controversy remains regarding if I-R injury exists in humans. While experiments required to definitively demonstrate I-R injury in humans are unethical and therefore impractical, a preponderance of evidence indicates that the same biochemical pathways mediating I-R injury in numerous pre-clinical models each exist in human [19–21]. Targeting I-R injury has been a focus of cardiovascular research for more than 30 years, and a large number of approaches have been shown to reduce I-R injury in preclinical models of AMI [22]. However, numerous trials trying to replicate those findings in the clinical setting have failed [23]. These approaches have included various pharmacotherapies, device-mediated interventions, and myocardial cooling [23–26].

Several possible explanations for the failure of these pre-clinical studies to translate into clinical benefit have been postulated. First, the animal models used to investigate various therapies have well-established shortcomings and fail to replicate the intricacies of human coronary artery disease including the typical array of comorbid conditions and background medical therapies present in patients. Second, to be effective, drugs that target the ischemic myocardium require access to the affected myocardium. During AMI, however, perfusion to the ischemic myocardium is by definition minimal if existent at all. Due to the overall health of animals used in preclinical studies, intact collateral flow (though physiologically limited in many animal models) may allow for more efficient delivery of these agents which is otherwise limited or impossible in real-world human subjects. Lastly, human subjects are often chronically exposed to background medical therapies to address on-going medical issues. These drugs can interact with the same cellular protein or molecular mechanism that is targeted by the developed therapy, thereby altering the expected response. A good example of this is the widespread use of P2Y12 inhibitors. These drugs (like ticagrelor, clopidogrel, or cangrelor) inhibit platelet activation and are routinely given to patients suffering an AMI in order to prevent thrombosis. P2Y12 inhibitors have been demonstrated to activate cardioprotective signal transduction pathways [27]. Activation of these very same pathways are often the end targets of experimental interventional approaches like pre- and post-conditioning, and numerous pharmaceutical therapies [28, 29]. The expected effect of these interventions on limiting infarct scar size (which was observed in pre-clinical trials where P2Y12 inhibitors were not present) has yet to materialize in clinical trials. This could, at least in part, be due to this targeted pathway already being activated in both the experimental and control arms.

It is worth noting here that targeted therapies (like pharmaceutical and gene therapy approaches) by design target a single protein or a small family of functionally related proteins in the myocardium in order to limit infarct size. Apoptosis, fibrosis, and other maladaptive ventricular remodeling processes are mediated by numerous, complex, and interacting biochemical and/or genetic pathways. These may be fundamentally impossible to regulate by targeting single proteins or genes or even a relatively focused family thereof. This complexity is reflected in the sheer number of proteins and genes with altered expression or regulation in the diseased heart [30–32], and these changes begin in the acute setting early in the index MI [33, 34]. While not without some successes, targeted therapies as a paradigm to minimize infarct scar formation may ultimately be a limited or even a failed concept. A treatment paradigm that alleviates the primary stressor itself and not just a single downstream component or biochemical pathway may be required. Acute cardiac unloading is a new approach with pleiotropic effects that may combine to minimize infarct scar size and limiting I-R injury.

## Acute Cardiac Unloading

Acute cardiac unloading is any maneuver, therapy, or intervention that decreases the power expenditure of the ventricle and limits the hemodynamic forces that lead to ventricular remodeling after insult or injury to the heart [35, 36]. Acute ventricular unloading using percutaneous ventricular assist devices (pVADs) is emerging as a clinically viable strategy to limit cardiac power expenditure, protect against I-R injury, promote myocardial salvage, limit infarct size, and attenuate ventricular remodeling in the setting of AMI. In vivo and ex vivo experiments dating back 40 years have repeatedly demonstrated that unloading the ventricle before, during, or after AMI favorably affects cardiac function post-infarction [37–40]. However, until recently, achieving ventricular unloading in the clinical setting was not technically feasible. No drug or medical device could effectively unload the heart while, at the same time, maintain or improve CO and blood pressure. Accordingly, this approach to treating AMI was never developed. This changed in the early 2000s.

With the advent of miniaturized pVADs like the Impella (Abiomed, Inc., Danvers, MA, USA) or the TandemHeart (LivaNova, Mirandola, Italy), safe and effective ventricular unloading became clinically possible and research was reinvigorated. Percutaneous mechanical blood pumps offer the unique ability of decreasing the power expenditure of the heart by supplanting the need of the heart to pump blood. This decreases the workload of the heart and unloads it, reduces its metabolic demands, and offsetts the physical forces that contribute to remodeling during the critical period of acute myocardial injury and post-infarct inflammation.

The Impella family of devices and the TandemHeart devices are the only pVADs currently available in the clinic. Each provides hemodynamic support, and both unload the heart [41, 42]. Peripheral ECMO, though able to support end-organ perfusion and maintain perfusion pressure, does not unload the heart [35]. In hemodynamic simulations, animal models, and humans, ECMO has been shown to actually increase the load on the heart and often requires an additional intervention to unload the left ventricle [43–46]. The intra-aortic balloon pump (IABP) may provide a small improvement in diastolic coronary perfusion, but it does not significantly augment CO nor unload the LV and therefore leaves the heart under significant stress [43].

After placement within the heart, pVADs actively remove oxygenated blood from the heart and return it directly to the systemic circulation. In this way, CO and mean arterial pressure (MAP) are maintained through a mechanical means independent of cardiac function. The Impella 2.5, CP, and 5.0 are LV-to-Ao pumps, and they directly unload the LV in parallel with physiological flow by pumping blood directly from the LV into the ascending aorta [41]. This differs from the TandemHeart device which is an LA-to-arterial pump that secondarily unloads the LV in parallel to physiological flow by removing blood from the left atria (LA) and pumping it to the iliac artery for distribution into the arterial system. Despite differing hemodynamic effects, the Impella and TandemHeart have been demonstrated to effectively reduce the work of the LV while improving systemic blood flow [43, 47–50].

## Unloading Reduces MVO2 and Infarct Scar Size

**Fig. 1:**
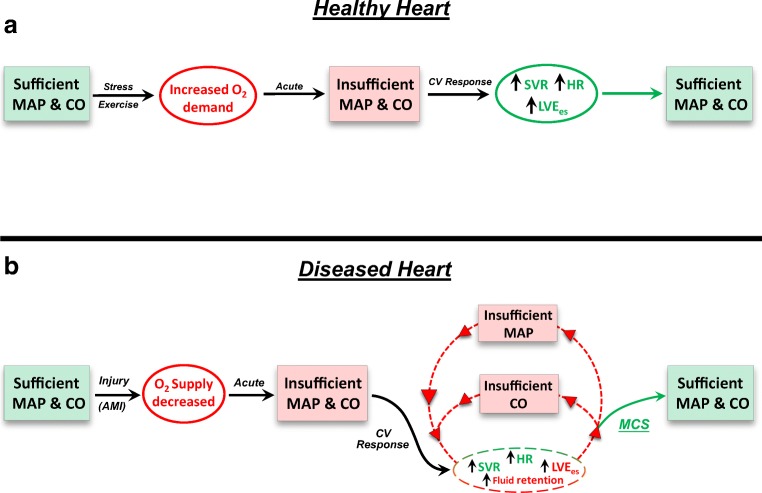
Schematic of the cardiovascular response to maintain mean arterial pressure and cardiac output in a healthy and diseased heart. **a** In the healthy heart, exercise or stress causes an acute increase in O_2_ demand by the body. Baseline levels of MAP and CO are insufficient to meet this demand. This prompts acute responsive mechanisms which collectively increase CO and maintain sufficient MAP. **b** In a diseased or injured heart, such as after an AMI, O_2_ supply is impaired due to damage of the heart, and MAP and CO subsequently decrease. If the damage is too large, cardiovascular responsive mechanisms may be unable to increase MAP and CO to sufficient levels, leading to a feedback loop that places the heart under increased stress due to chronically insufficient MAP and CO. Initiation of mechanical circulatory support (MCS) via a pVAD like the Impella unloads the ventricle and augments MAP and CO, thereby relieving the stress placed on the heart under these conditions: MAP, mean arterial pressure; CO, cardiac output; SVR, systemic vascular resistance; LVE_es_, slope of the left ventricular end-systolic pressure volume relationship (contractility)

The primary function of the heart is to provide blood flow to meet the metabolic demands of the body. The healthy heart is well-equipped to manage the task, such as during exercise (Fig. 1a). However, during AMI, the functional capacity of the heart is compromised by the loss of muscle leading to decreased systemic O_2_ supply and reduced CO and MAP. Consequently, the remaining viable myocardium must work harder to maintain end-organ perfusion. In response to insufficient MAP and CO, the cardiovascular system activates a series of neural reflex and humoral reactions, such as the baroreceptor reflex and fluid retention mechanisms. Often, these compensatory mechanisms are sufficient to restore CO and MAP. However, if the extent of damage to the heart is large enough, it will be physically incapable of increasing MAP and CO to sufficient levels. This leads to a feedback loop in which more and more stress is placed upon an already injured heart (Fig. 1b) [51]. Without intervention, this stress will inevitably precipitate further myocardial damage and dysfunction. The loss of viable myocardium shifts the burden of CO onto a smaller and smaller muscle mass, significantly increasing the power expenditure of the remaining myocardium. Mechanical circulatory support (MCS) via a pVAD acutely unloads the heart of its mechanical workload while augmenting both MAP and CO. In this way, patient hemodynamics are stabilized, staving off circulatory collapse.

**Fig. 2:**
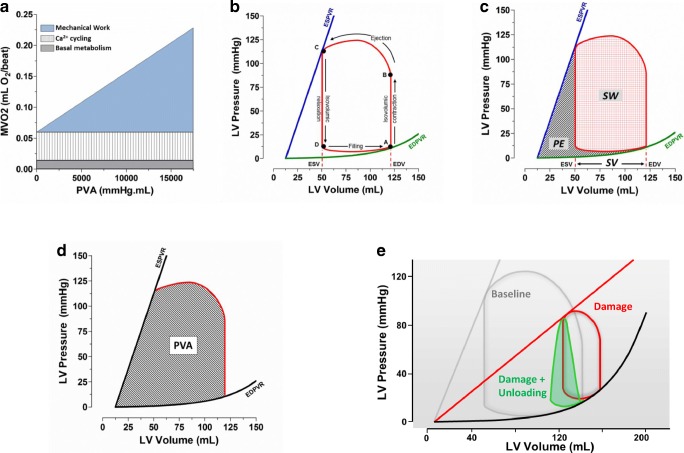
Pressure-volume analysis for assessment of ventricular mechanics and energetics. **a** Components of myocardial oxygen consumption. The pressure-volume area (PVA, mmHg·mL) quantifies the total mechanical work of the heart per beat and is linearly related to myocardial oxygen consumption (MVO2) [54]. MVO2 of the completely unloaded heart (i.e., at PVA = 0) is determined by basal metabolism and energy for calcium cycling and other pumps associated with ion fluxes. The PVA-dependent component of MVO2 relates to energy for cross bridge cycling. **b** A single idealized pressure-volume loop demonstrating the events of the cardiac cycle from end-diastole (A), though opening of the aortic value (B), end-systole (C), through the onset of diastolic filling (D). **c** The total mechanical work performed during a single beat is made up of the stroke work (SW) and the potential energy (PE; energy not contributing to the pumping of blood). **d** The total mechanical work of the heart is estimated by the total of SW and PE, and is called the pressure-volume area (PVA). **e** The effect of acute myocardial damage right-shifts the pressure-volume loop and decreases the slope of the ESPVR, reflective of a loss of myocardial mass and or contractility (due to ischemia and infarction). Increased filling pressures following myocardial damage increases end-diastolic volume (red loop). The effect of ventricular unloading by a ventricular assist device left-shifts the pressure-volume loop towards lower volumes (green loop), reflecting pressure and volume unloading. The PVA of the green loop is smaller, indicating less work being performed by the heart per beat

It has been demonstrated in multiple preclinical models that infarct size varies directly with myocardial oxygen consumption (MVO2) [52]. The smaller the MVO2, the smaller the resultant infarct scar. MVO2 reflects the total amount of work performed by the heart. In the simplest terms, total mechanical work of the heart along with energy required for calcium cycling make up the majority of total MVO2 with a smaller but constant amount utilized for basal metabolism [36, 43] (Fig. 2a). Heart rate (HR) and myocardial contractility are also major determinants of MVO2. At higher heart rates, there is greater MVO2 per minute. Myocardial contractility is typically mediated by changes in calcium cycling. Increased calcium means increased energy needs for this process, and the MVO2-PVA relationship shifts upwards. That means, the heart consumes more oxygen to perform the same amount of work at a higher contractility. This may contribute to the poor outcomes associated with the use of inotropes to support cardiogenic shock patients.

Total mechanical work, and thus MVO2, can be quantified through pressure-volume (P-V) analysis. The P-V loop is the dynamic relationship between instantaneous ventricular pressure and volume during a single heartbeat (Fig. 2b) [53]. The P-V loop is bound by the end-systolic pressure-volume relationship (ESPVR) on top and the end-diastolic pressure volume relationship (EDPVR) on the bottom. The ESPVR defines the maximal pressure able to be developed at a given volume, and the slope of this line (LVE_es_) is an index of contractility. The EDPVR defines the passive properties of the myocardium (i.e., ventricular compliance). The area inside the loop quantifies the external stroke work (SW) of the heart which is the mechanical energy used to pump blood (measured in mmHg·mL, i.e., a Joule). The remaining area bound by the ESPVR, the EDPVR, and the isovolumic relaxation portion of the P-V loop represents the potential energy (PE) that resides in the myofilaments at the end of systole that was not transduced into external work (Fig. 2c). The pressure-volume area (PVA) is the sum of these two areas (SW + PE) and quantifies the total mechanical energy expenditure of each beat of the left ventricle (Fig. 2d). The relationship of MVO2 to PVA has been shown to be linear; PVA therefore provides a useful index of MVO2 [54]. Any intervention or therapy that diminishes the PVA would, therefore, also diminish MVO2.

Comparing the gray and red P-V loops in Fig. 2e, the difference between a healthy and damaged heart can be appreciated. In this simulated data [55], AMI leads to a loss of muscle and contractile reserve (echoed in the decreased LVE_es_, red loop), diminished ejection fraction and volume overload (seen in the rightward shift along the volume axis, and resulting from an increase in stressed blood volume from 1200 to 1600 mL), and a decrease in stoke volume of ~ 50%. In this state, the damaged heart must beat twice as fast in order to maintain the same CO, which is a tremendous stress. The myocardium also becomes increasingly inefficient at pumping blood. This is reflected in the disproportional increase in PE and reduced SW making up the PVA. In short, the heart is working harder but accomplishing less.

Interventions for AMI that are currently used in the standard of care are unable to efficiently or safely decrease MVO2. The central problem is that these interventions do not uncouple ventricular load from the requirement of heart to pump blood and support end-organ perfusion. Inotropes, vasopressors, and counterpulsation each requires the heart to work harder, and this increases MVO2. Indeed, use of inotropes in the treatment of AMI has been plagued by increased incidence of arrhythmia, hypotension, and myocardial ischemia [56–59]. Along with inotropes, vasopressors are routinely used to treat AMI complicated by refractory cardiogenic shock (AMICS) [60]. Vasopressors increase the afterload the damaged heart must pump against, and this increases MVO2 as well. They have been linked to ventricular arrhythmias, contraction-band necrosis, and infarct expansion [61, 62]. Lastly, the randomized controlled IABP-SHOCK II trial failed to show any clinical benefit for the use of the IABP in patients with AMICS and may, in fact, cause harm [63]. Like expecting a sprinter to rehabilitate from a hamstring injury while always at full sprint, current approaches never afford the heart the opportunity to reduce its workload so it can rest and recover from damage.

By supplanting blood pumping requirements, pVADs minimize MVO2 and maximize the opportunity to reduce metabolic demands of the heart. The green P-V loop in Fig. 2e demonstrates how SW and ventricular volume are minimized in the acutely unloaded heart. In this simulated data, a transvalvular pump (such as the Impella CP) is set to 3.5 L/min (maximal support for this pump). This yields significant decreases in PVA and MVO2 [64]. Furthermore, mechanical support increases CO and MAP. As a consequence, adrenergic tone is relieved and HR may subsequently fall [44]. As HR is a major contributor to MVO2 per minute, its attenuation significantly decreases oxygen demand. Multiple independent pre-clinical studies in varying species have each demonstrated that short term use of pVADs in the setting of AMI unloads the heart, significantly decreases MVO2, and limits infarct scar size compared to reperfusion alone [33, 47, 64–67].

## Acute Cardiac Unloading and I-R Injury

**Fig. 3:**
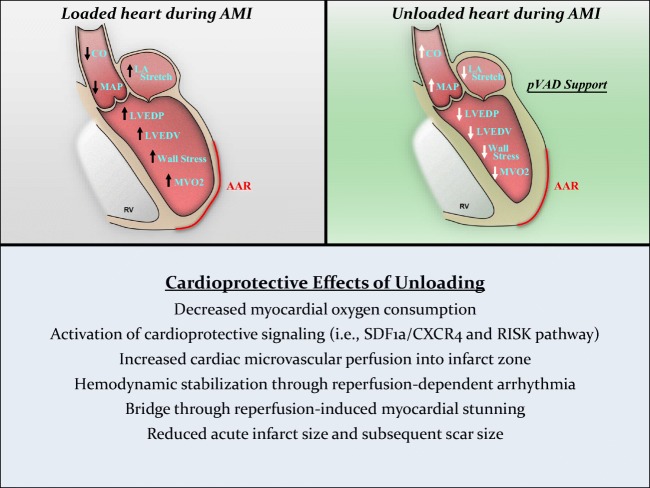
Central figure. The cardioprotective mechanisms of acute ventricular unloading*.* After AMI, CO and MAP drop due to ischemic damage to the myocardium in the area at risk (AAR) resulting in hemodynamic derangement, and increased O_2_ consumption and wall stress (upper right). Acute ventricular unloading by a pVAD like the Impella attenuates these hemodynamic effects by aspirating blood out of the ventricle directly into the aorta, thereby restoring CO and MAP. This results in decreased MVO2 and wall stress (upper left). Acute ventricular unloading prior to reperfusion mitigates ischemia-reperfusion injury and provides cardioprotection through multiple mechanisms (bottom)

Myocardial I-R injury impacts at least four different aspects of myocardial physiology: (1) lethal myocardial reperfusion injury and infarct expansion, (2) reperfusion-induced arrhythmias, (3) microvascular obstruction, and (4) myocardial stunning [22]. Mounting clinical and pre-clinical evidence indicates that each of these effect of I-R injury may be independently attenuated by acute cardiac unloading (Fig. 3, central figure).

### Lethal Myocardial Reperfusion Injury

Chief among the effects of I-R injury in terms of affecting infarct scar size are lethal myocardial reperfusion injury and infarct expansion. Reperfusion injury is defined as reperfusion-dependent death of cardiomyocytes that were viable at the time reperfusion occurred [68]. The underlying molecular mechanisms of lethal reperfusion injury have been extensively reviewed elsewhere [22, 69]. Decades of research has demonstrated that activation of the cardioprotective RISK signaling pathway attenuates reperfusion and limits infarct scar size.

Seminal pre-clinical work by Kapur and colleagues has demonstrated that acute unloading by the Impella device introduced prior to reperfusion activates stromal cell-derived factor (SDF-1α)-dependent cardioprotective signaling [33, 70]. SDF-1α acts through its cognate receptor, CXCR4, to mediate myocardial protection and salvage by activating Erk1/2 and Akt signaling, while simultaneously inhibiting glycogen synthase kinase (GSK)-3β. The SDF-1α/CXCR4 signaling axis has been previously linked with I-R injury protection and myocardial salvage [71, 72]. Acute unloading prior to reperfusion was also demonstrated to limit apoptotic signaling through effects on BCL-2, BAX, and BCL-CL, and the maintenance of mitochondrial integrity [33]. Importantly, these biochemical effects limited infarct scar size in both the acute and chronic (28-day post-MI) phase.

The effects of ventricular unloading on infarct scar size extend beyond cardioprotective signaling. Clinical and preclinical data have demonstrated that acute unloading decreases ventricular wall stress, an expected outcome resulting from pressure and volume unloading [47, 64, 66, 73–75]. Early critical work conducted in the 1970s and 1980s linked acutely increased wall stress and filling pressures with the disruption of connective tissue within the heart [76–79]. This effect on the architecture of the heart and the organization of the extracellular matrix underlies infarct expansion, the disproportionate thinning, and dilatation of acutely infarcted myocardium [77–79]. Infarct expansion primarily occurs in the acute phase of post-MI ventricular remodeling (< 24 h) and leads to early cardiac dilatation. Limiting infarct expansion reduces final infarct scar circumference and attenuates maladaptive ventricular remodeling [80].

While a decreased absolute number of myocytes, smaller myocyte cross sectional area, decreased capillary density, and infarct compaction plays a role in infarct expansion, the predominant mechanism of infarct expansion is side-to-side slippage of myocyte bundles in the ventricular wall [79]. Slippage results from the proteolysis and/or mechanical shearing of connective tissues within the extracellular matrix (ECM; i.e., collagens, gelatin, laminins, etc.). In this damaged framework, the structural integrity of the myocyte bundles is compromised, and they physically rearrange, resulting in thinning of the infarcted myocardium, chamber dilation, and an acute decrease in cardiac function [76, 78, 79, 81].

Matrix metalloproteinases (MMPs) are proteolytic enzymes that degrade components of the ECM and are directly involved in regulating its composition [82]. MMP activity is increased in the post-MI heart and plays an important role in ventricular remodeling and chamber dilatation [83]. New data from the Kapur lab has shown that acute ventricular unloading decreases MMP-2 and MMP-9 activity in an AMI model, leading to smaller infarct scar size [33]. This posits an intriguing possibility that acute unloading may limit ECM degradation. While the direct relationship has not been specifically investigated, the combined effects of ventricular unloading on wall stress and MMP activity may limit infarct expansion, in part, explaining the observed effect of unloading on limiting final infarct scar size.

In line with limiting infarct expansion, previous studies have indicated that reduced LV wall stress limits MVO2 and PVA, linking it with infarct scar size [84, 85]. Indeed, increased wall stress is an independent predictor of post-discharge heart failure after AMI [86]. Lastly, calcium handling mechanisms are disrupted during AMI and contribute to I-R injury and infarct scar size. Wei et al. [66] demonstrated that acute unloading prior to reperfusion normalizes calcium handling in the post-MI heart. They showed that these effects were maintained 12 weeks post-MI, and ultrastructural damage to the heart was ameliorated by acute unloading, indicating an effect of unloading on the ECM.

### Reperfusion-Induced Arrhythmia

Clinical data has demonstrated that patients undergoing primary coronary intervention (PCI) often experience ventricular arrhythmia at the point of reperfusion [87]. While these arrhythmias are usually medically managed or terminated on their own, they do put the patient at increased risk for morbidity. Myocardial stretch is well-known to increase arrhythmogenicity. Acute unloading of the heart via initiation of pVAD support maintains CO while relieving ventricular wall stress and stretch. This may minimize the risk of adverse effects associated with reperfusion-induced ventricular arrhythmias. The ability of the Impella to safely bridge a patient through ventricular dysfunction was clearly demonstrated by Verma et al. [88] in a high-risk patient undergoing PCI. This clinical report verified the ability of the Impella device to maintain CO and perfusion pressure despite a non-pulsatile LV. Furthermore, several reports demonstrate Impella support safely bridging patients through ventricular tachycardia ablation procedures, acute right ventricular failure, and even cardiac arrest [89–92]. In a canine model of acute decompensated HF, Kawashima et al. [44] demonstrate that Impella support led to superior ventricular unloading compared with ECMO, and hearts supported by Impella had a higher rate of successful defibrillation and recovery of sinus rhythm. New data in an animal model of subacute HF has provided the first mechanistic insights into how ventricular unloading may limit arrhythmia formation. Ishikawa et al. [93] demonstrate that LV unloading by the Impella leads to passive unloading of the left atria and subsequent protection from atrial fibrillation by limiting atrial stretch. They demonstrate that this relieves atrial wall stress and attenuates post-MI NADPH oxidase overexpression and diminishes ryanodine receptor phosphorylation. While these particular findings focus on atrial arrhythmogenic mechanisms, they demonstrate that the effect relieving myocardial wall stress extends beyond hemodynamics and has direct molecular effects at the level of the single myocyte. Collectively, these data demonstrate that pVAD support limits arrhythmogenesis and can maintain CO and MAP until native heart function and sinus rhythm can be recovered.

### Microvascular Obstruction

Krug et al. [94] defined microvascular obstruction post-MI as the inability to reperfuse a previously ischemic region , and Kloner et al. [95] demonstrated that the no reflow phenomenon was associated with specific ultrastructural abnormalities of the microvasculature, including focal endothelial protrusions and swelling that blocked the lumen of small vessels. A number of other structural and molecular components also contribute to this phenomenon. Capillary damage leading to impaired auto-regulation, microvascular, and coronary compression resulting from increased ventricular wall stress, micro-embolization and microthrombus, and neutrophil plugging each play a role. Several experimental and clinical studies show that the presence of no reflow is associated with adverse LV remodeling including thinner infarct scar and more LV dilatation. The authors direct the interested reader to recent extensive reviews on this phenomenon [96, 97].

Ventricular volume increases during infarction, leading to increases in LV pressure (see Fig. 2e). This places a significant outward force on the ventricular wall increasing subendocardial wall stress. This wall stress acts as a compressive force on the coronary arteries and vasculature, increasing the resistance to flow. Using the current standard of care, even after reperfusion is established, wall stress remains high and impaired microvascular flow persists [98]. This exacerbates ischemia, leading to further cell damage and cell death. Numerous reports have demonstrated that unloading the ventricle with the Impella decreases ventricular wall stress, and the expected increase in coronary flow is observed [47, 70, 73, 98–100]. A recently published report found that ventricular unloading decreases wall stress and leads to a near-doubling of regional blood flow within an established infarct zone [73]. These data indicate that acute unloading is able to modulate microvascular flow by affecting wall stress even where auto-regulatory mechanisms are impaired. Furthermore, recent data indicate that unloading limits MMP activity, which would be expected to decrease both inflammation and maladaptive ventricular remodeling (see above) [33, 101]. These data open the possibility that unloading may mediate neutrophil activity and potentially attenuate neutrophil plugging, although this effect remains currently unexplored. Together, these reports indicate that acute cardiac unloading may limit microvascular obstruction in the setting of AMI.

### Myocardial Stunning

Myocardial stunning is the reversible contractile dysfunction present after reperfusion that is not associated with tissue damage. It results from oxidative stress and intracellular Ca^2+^ overload that develops within cardiomyocytes during ischemia [102, 103]. Contractile dysfunction associated with stunning manifests itself upon reperfusion in the form of non-contractile myocardium. While stunning is reversible, it can take several days to weeks to resolve [104]. The temporarily impaired function places the patient at increased risk, especially those with pre-infarct LV dysfunction or unresolved coronary artery disease. Circulatory support by a pVAD could bridge patients through or even hasten recovery from stunning, thereby minimizing the associated risks and maintaining patient hemodynamics until native heart function can be recovered. Clinical and preclinical data suggest that two independent mechanisms of ventricular unloading and hemodynamic support may mitigate myocardial stunning. The first is reducing myocardial stress. Pharmacologically reducing myocardial stress by lowering blood pressure (decreased afterload) via Ca^2+^ channel blockers or ACE-inhibitors has demonstrated efficacy of improving contractile function of stunned myocardium [105–108]. HR reduction (a key aspect of unloading) by ivabradine also hastens recovery from stunning, but beta-blockade by atenolol which would be expected to unload the heart had no effect [109]. This emphasizes the difficulties often encountered by using targeted pharmacological approaches as discussed above.

Second, increased perfusion pressure can impact myocardial stunning. Early data in patients supported with ECMO post-AMICS demonstrated hastened recovery from myocardial stunning [110]. While ECMO is not an unloading pump, this data demonstrates that increased perfusion pressure may mitigate the effects of stunning, likely through increased coronary blood flow. Taken together, these data suggest that a pVAD, like the Impella, that both decreases myocardial stress while simultaneously supplying increased hemodynamic support will be able to hasten recovery from stunning. Furthermore, the effect of unloading of increasing microcirculatory blood flow just discussed is an additional mechanism by which unloading may promote a quicker recovery from stunning. This hypothesis emphasizes the pleiotropic nature of acute ventricular unloading. However, this has not been directly tested, and further investigations are required.

## Future Uses of Acute Unloading

Acute unloading offers a unique platform on which other therapies may be built. Particularly appealing is the possibility of using mechanical support to bridge patients through the otherwise intolerable or high-risk doses of certain pharmaceutical interventions. An excellent example of this is the limitation of β-blocker therapy to treat infarct scar size in the setting of cardiogenic shock. Clinical studies have shown the potential effectiveness of β-blockers [20, 111], and this class of drug is indicated when hemodynamic conditions are stable. However, use is contraindicated in cardiogenic shock because it may further diminish CO and MAP, placing the patient at excessive risk for hemodynamic collapse. In this setting, mechanical circulatory support could be used to maintain CO and MAP at sufficient levels and potentially allow for safer dose escalation of β-blocker therapy. Given the important role of HR and myocardial contractility on MVO2, this drug-device combination has great potential. In fact, a preclinical model of AMI showed that the combination of a pLVAD with either ivabradine (a drug that reduces HR) or with vagal stimulation (which also lowers HR) reduced infarct size while maintaining systemic perfusion [67, 112]. The efficacy of drug-device combinations has not yet been tested clinically.

## Conclusion

The potential clinical applications of acute ventricular unloading are only now being explored, thanks to advent of safe and effective temporary circulatory devices like the Impella pump. Data from the past decade have demonstrated how acute unloading acts in a pleiotropic manner when in the setting of AMI. We now know that acute unloading simultaneously decreases MVO2, maintains sufficient CO and MAP, activates cardioprotective signaling, decreases ventricular and atrial wall stress, increases coronary and microvascular blood flow, and can safely bridge patients through cardiac arrest and/or arrhythmia. When mechanical support is applied prior to reperfusion all of these independent, yet single device-dependent, effects combine to limit infarct scar size post-AMI and minimize procedural risks to the patient. The clinical potential of acute unloading prior to reperfusion to limit infarct scar size and attenuate the development of HF post-AMI is the focus of the current Door-To-Unload clinical trial (www.clinicaltrials.gov, NCT03000270) [113, 114]. Research in this new field is accelerating, yet much remains to be explored. Further understanding of the molecular and physiological mechanisms mediating the cardioprotective effects of acute unloading will likely yield exciting results and expose new avenues for research.

## Abbreviations

AMI: Acute myocardial infarct
AMICS: Acute myocardial infarct complicated by cardiogenic shock
CO: Cardiac output
DTB: Door-to-balloon
ECM: Extracellular matrix
ECMO: Extracorporeal membranous oxygenation
EDPVR: End-diastolic pressure-volume relationship
ESPVR: End-systolic pressure-volume relationship
HF: Heart failure
HR: Heart rate
IABP: Intra-aortic balloon pump
I-R: Ischemia-reperfusion injury
LA: Left atria
LV: Left ventricle
LVE_es_: Slope of the left ventricular end-systolic pressure volume relationship
MAP: Mean arterial pressure
MCS: Mechanical circulatory support
MI: Myocardial infarct
MMP: Matrix metalloproteinase
MVO2: Myocardial oxygen consumption
PCI: Percutaneous coronary intervention
PE: Potential energy
P-V: Pressure-volume
PVA: Pressure-volume area
pVAD: Percutaneous ventricular assist device
RISK: Reperfusion injury salvage kinase
SDF1-α: Stromal cell-derived factor 1α
SW: Stroke work
